# Supplementary material for: Incidence and Survival Outcomes of Colorectal Cancer in Long-Term Metformin Users with Diabetes: A Population-Based Cohort Study Using a Common Data Model
Source: J Pers Med. 2022 Apr 5;12(4):584. doi: 10.3390/jpm12040584 (PMC9031185; doi:10.3390/jpm12040584)

Table S1. List of negative control outcomes

Concept ID	Concept Code	Concept Name
378256	46670006	Abnormal reflex
440424	87486003	Aphasia
439237	52684005	Assault
378424	82649003	Astigmatism
261880	46621007	Atelectasis
134118	400190005	Atrophic condition of skin
4224118	40492006	Bladder dysfunction
80509	203465002	Bone cyst
434626	20010003	Borderline personality disorder
438407	78004001	Bulimia nervosa
134765	238108007	Cachexia
4172458	49883006	Candidiasis of skin
436740	17382005	Cervical incompetence
381581	1482004	Chalazion
4307254	423125000	Closed fracture
4047787	123971006	Colles' fracture
198075	240542006	Condyloma acuminatum
73302	64217002	Curvature of spine
4242416	58588007	Cutis laxa
433163	238107002	Deficiency of macronutrients
4047269	229844004	Deformity of foot
133228	80967001	Dental caries

4147672	30415006	Disease due to Papilloma virus
4153380	371160000	Disorder of carotid artery
4140510	3305006	Disorder of lymphatic vessel
433440	78667006	Dysthymia
376132	62909004	Ectropion
440695	302690004	Encopresis
438872	267023007	Excessive eating - polyphagia
78804	27431007	Fibrocystic disease of breast
4131595	12676007	Fracture of radius
74855	33839006	Genital herpes simplex
441788	240532009	Human papilloma virus infection
76737	55434001	Hydrocele
4029582	237793004	Hyperandrogenization syndrome
195212	47270006	Hypercortisolism
438134	77692006	Hypersomnia
140362	36976004	Hypoparathyroidism
4322737	427898007	Infection of tooth
4207688	55184003	Infectious enteritis
79072	266579006	Inflammatory disorder of breast
139099	400097005	Ingrowing nail
4288544	396232000	Inguinal hernia
444191	125593007	Injury of face
444130	125604000	Injury of foot
134222	125597008	Injury of forearm

4029966	128609009	Intracranial aneurysm
437409	127296001	Intracranial injury
4297984	76844004	Local infection of wound
4018050	10443009	Localized infection
439840	1415005	Lymphangitis
4163232	45198002	Mastitis
440389	91138005	Mental retardation
436100	60380001	Narcolepsy
4262178	397732007	Neurogenic dysfunction of the urinary bladder
193874	8009008	Nocturnal enuresis
4171549	419153005	Nodular goiter
442274	52073004	Oligomenorrhea
4215978	414941008	Onychomycosis
4171915	274718005	Orchitis
380731	3135009	Otitis externa
378160	65668001	Otorrhea
192606	60389000	Paraplegia
253796	36118008	Pneumothorax
195501	69878008	Polycystic ovaries
4153877	269406001	Post-traumatic wound infection
434319	44001008	Premature ejaculation
373478	41256004	Presbyopia
199876	73998008	Prolapse of female genital organs
4295888	76641005	Prolapse of intestine

194997	9713002	Prostatitis
4146239	267802000	Pruritus of genital organs
4285569	68633000	Pupillary disorder
81336	57773001	Rectal prolapse
380395	314407005	Retinal dystrophy
141825	267369002	Simple goiter
137054	201066002	Skin striae
434630	3745000	Sleep-wake schedule disorder
4195698	67801009	Tenosynovitis
4339088	87860000	Testicular mass
133141	6020002	Tinea pedis
440814	70070008	Torticollis
435140	67426006	Toxic effect of alcohol
4270490	62994001	Tracheitis
4028970	13617004	Tracheobronchitis
193326	87557004	Urge incontinence of urine
4092565	24976005	Uterine prolapse
140641	57019003	Verruca vulgaris
197036	197811007	Vesicoureteric reflux
261326	75570004	Viral pneumonia

Figure S1. Covariate balance before and after propensity matching

Blue dots indicate the SMD of each covariate between the target and comparative cohorts before and after propensity-score matching. SMD, standardized mean difference; PS, propensity score

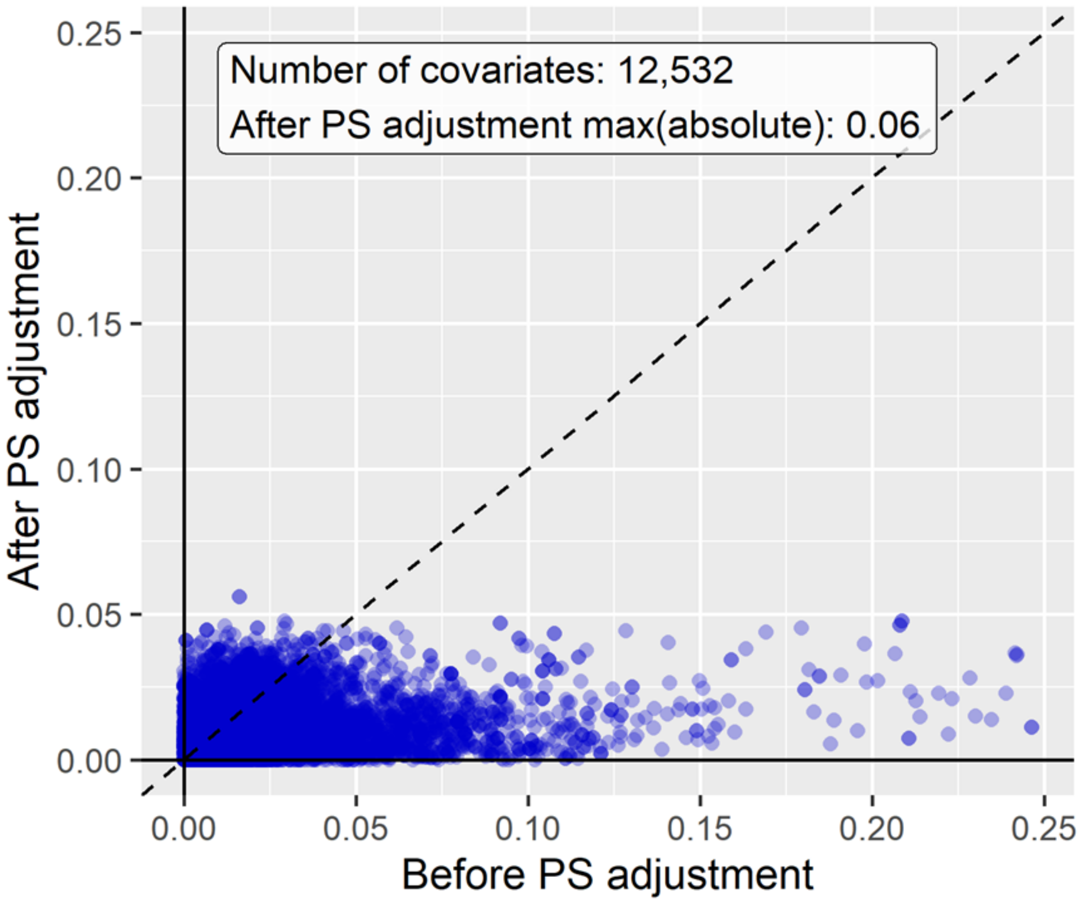

Figure S2. A plot of calibrated significance testing in the analysis

Blue dots indicate the estimates for negative control outcomes. The estimates in the orange areas have a *P*<0.05 based on a calibrated *P*-value calculation.

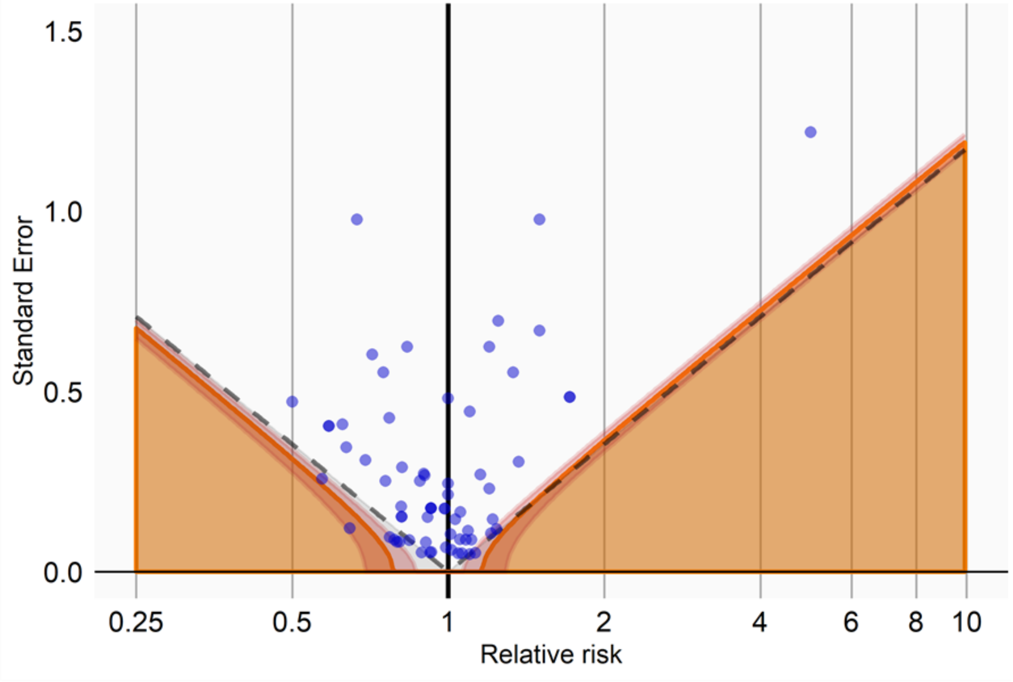

Supplement: Supplementary file 1 [file jpm-12-00584-s001.zip › jpm-1610821-supplementary.pdf]
